# Transplacental transmission of *Theileria orientalis* occurs at a low rate in field-affected cattle: infection *in utero* does not appear to be a major cause of abortion

**DOI:** 10.1186/s13071-017-2166-9

**Published:** 2017-05-08

**Authors:** Emma Swilks, Shayne A. Fell, Jade F. Hammer, Narelle Sales, Gaye L. Krebs, Cheryl Jenkins

**Affiliations:** 1 School of Animal and Veterinary Sciences, Charles Sturt University, Locked Bag 588, Wagga Wagga, NSW 2678 Australia; 2 NSW Department of Primary Industries, Elizabeth Macarthur Agricultural Institute, Woodbridge Rd, Menangle, NSW 2568 Australia; 3 Main Street Veterinary Clinic, 325 Main Street, Bairnsdale, Vic 3875 Australia

**Keywords:** *Theileria orientalis*, Transplacental transmission, Theileriosis, qPCR, Serology

## Abstract

**Background:**

Bovine theileriosis, caused by the haemoprotozoan *Theileria orientalis*, is an emerging disease in East Asia and Australasia. Previous studies have demonstrated transplacental transmission of various *Theileria* spp. but molecular confirmation of transplacental transmission of *T. orientalis* has never been confirmed in the field. In this study, cow-calf (< 48 h old) pairs were sampled across 3 herds; opportunistic samples from aborted foetuses or stillborn calves were also examined. Molecular (multiplex qPCR) and serological (ELISA) methods were used to determine infection prevalence and the presence of anti-*Theileria* antibodies in each herd. In addition, pregnant heifers and foetal calves were sampled at abattoir and tested for the presence of *T. orientalis* by qPCR.

**Results:**

The qPCR results indicated that, even though there was a high prevalence of *T. orientalis* infection in cows, the rate of transplacental transmission to their calves was low, with only one newborn calf from one herd and one foetus from the abattoir testing positive for *T. orientalis* DNA. Five aborted foetuses and stillborn calves, 3 of which were derived from a herd experiencing a high number of clinical theileriosis cases at the time of sampling, all tested negative for *T. orientalis* by qPCR. This suggests that *in utero* infection of calves with *T. orientalis* may not be a major driver of abortions during theileriosis outbreaks. Temporal monitoring of 20 calves born to *T. orientalis*-positive mothers indicated that *T. orientalis* was detectable in most calves between 10 and 27 days *post-partum*, consistent with prior field studies on adult cattle introduced to *Theileria*-affected herds. There was a positive correlation between the ELISA ratio of newborn calves and their mothers within 48 h of calving; however, maternal antibodies were only detectable in some calves and only for 4–4.5 weeks *post-partum*. All calves displayed high parasite loads peaking at 4–8 weeks *post-partum*, with only some calves subsequently mounting a detectable adaptive antibody response.

**Conclusions:**

These findings indicate transplacental transmission of *T. orientalis* appears to play only a minor role in persistence of *T. orientalis* infection in the field; however calves are highly susceptible to developing high level *T. orientalis* infections at 4–8 weeks of age regardless of whether maternal antibodies are present *post-partum*.

**Electronic supplementary material:**

The online version of this article (doi:10.1186/s13071-017-2166-9) contains supplementary material, which is available to authorized users.

## Background

In Australasia, bovine theileriosis is caused by the tick-borne haemoprotozoan, *Theileria orientalis* [1–4]. While this disease has been reported in East Asia for many years [4–6], the first definitive Australian cases of bovine theileriosis were detected in 2006 and were linked to a particular genotype of the parasite, *T. orientalis* Ikeda [1, 2, 7–9]. Since that time, the epizootic has spread to all mainland states of Australia and outbreaks have also been reported in New Zealand [3]. Other genotypes of *T. orientalis*, Type 1 (Chitose), Type 3 (Buffeli) and Type 5 are present in Australia but are generally described as benign [1, 8, 9].

Herd history and presenting clinical signs are often sufficient to arouse suspicion of theileriosis. The clinical signs of bovine theileriosis include lethargy, fever, anaemia and jaundice, with mortalities in naïve herds as high as 5%. Mortalities include still-born calves and late-term abortions, the latter being a major feature of this disease. Young calves also appear to be highly susceptible to theileriosis, with clinical disease and mortalities reported in herds where the disease is endemic [2, 10]. Infections are often subclinical; however, once cattle are infected, they appear to harbour the parasite long-term [11]. Stress appears to be a major factor in precipitating disease and therefore pregnant and recently calved cows are also at a high risk of developing clinical theileriosis [7, 12, 13]. *Theileria orientalis* is a vector-borne parasite with the ticks of the genus *Haemaphysalis* implicated in transmission in Japan [14–16], China [17], New Zealand [13] and Australia [18, 19]. While the sexual phase of the *T. orientalis* life-cycle occurs within the tick, mechanical transfer of the haploid erythrocytic phase has been shown to occur experimentally *via* lice in Japan [20], and can also occur by other mechanical methods [21]. Transplacental transmission of *T. orientalis* is of particular interest given the propensity of pregnant animals to abort and the susceptibility of young calves to disease. Prior studies indicate that transplacental transmission of other *Theileria* spp. can occur in their respective hosts including *T. equi* in horses [22, 23], *T. lestoquardi* in sheep [24] and rarely, *T. annulata* in cattle [25]. Transplacental transmission of *T. orientalis* was implicated in the infection and abortion of 100% of calves where the pregnant dams had been experimentally infected *via* ticks [26], while microscopic studies conducted in Japan suggested that transplacental transmission was occurring in field-affected cattle, but only at a low rate [27].

In Australian herds, infection with *T. orientalis* was detected *via* conventional PCR methods in calves as early as 1–2 weeks of age [2], but tick transmission could not be precluded. A more recent study detected *T. orientalis* on blood smears from calves between 4 and 20 days of age [10]; however these observations were not confirmed by molecular methods. In contrast, a recent study conducted on New Zealand herds experiencing outbreaks of disease, failed to detect transplacental transmission using a sensitive quantitative PCR technique [28]. Thus, despite recent severe outbreaks of bovine theileriosis in Australasia, it is currently unclear whether transplacental transmission contributes to late term abortion of calves and the persistence of this disease in affected herds. In this study we employed both molecular and serological techniques to investigate transplacental transmission of *T. orientalis* and determine whether infection *in utero* was a factor in calf abortion.

## Methods

### Herds

**Fig. 1:**
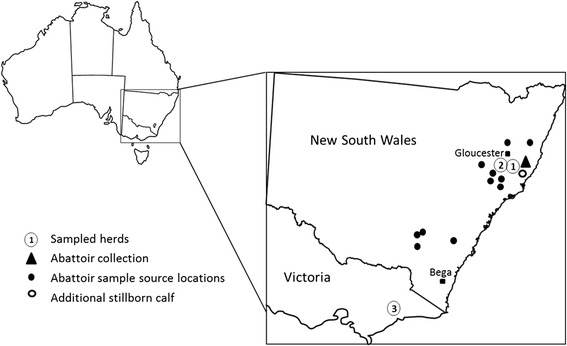
Locations of samples collected in this study

Three herds with a history of bovine theileriosis were examined in this study. Herd 1 consisted of 26 cow and calf pairs (Angus breed) and was located in the Gloucester area of New South Wales (NSW), where *T. orientalis* is known to be enzootic with a high prevalence [10]. Additionally, a sample of placental cotyledon, and one each of umbilical cord and cord blood were collected from one cow from Herd 1. Herd 2 consisted of Hereford cattle also from Gloucester NSW and contained 21 cows and 22 corresponding calves including one set of twin heifers. Herd 3 consisted of Holstein-Friesian cattle located at a dairy farm in Victoria which had recently been experiencing bovine theileriosis outbreaks and comprised 30 cow and calf pairs. Additional opportunistic samples from aborted (*n* = 2) and stillborn (*n* = 3) calves were collected. One sample was from a stillborn calf in Herd 1, one sample was from a property in Bungwahl, NSW near Gloucester and the remaining three samples were diagnostic submissions from a herd in Bega, NSW (Fig. 1) with 100% *T. orientalis* prevalence and which was experiencing clinical theileriosis cases at the time of sampling.

Finally, cow and foetal calf pairs were sampled at abattoir (Wingham, NSW). Nineteen EDTA bloods were collected from the cows at slaughter and 19 spleen samples were collected from the corresponding foetal calves post-slaughter. Samples collected at abattoir were collected from mixed beef breeds sourced from various herds throughout NSW (Fig. 1), and were selected on the basis of the cows being pregnant on day of sampling. Foetal calves sampled ranged from early to late term stages.

### Sample collection

All sampling was conducted between September 2014 and June 2015. Blood samples (EDTA and clotted blood) were collected from cows and calves in Herds 1–3 *via* either the jugular or caudal vein of the tail into sterile Vacutainer tubes. Samples from cows in Gloucester (Herds 1 and 2) were collected *pre-partum* [on average at 5 weeks prior to parturition, Time point 1 (TP1)]. Cows from Victoria (Herd 3) were sampled within 48 h of parturition [Time point 2 (TP2)]. Calves from all herds were sampled within 48 h of birth at TP2. Temporal sampling of 10 calves each from Herds 1 and 2 was undertaken at 3 and 7 days *post-partum* then weekly over a 4 week period *post-partum*, to determine the time to patency. Blood and/or spleen samples were also collected from opportunistic dead newborn, stillborn and aborted calves as part of clinical disease investigations. Tissues collected at abattoir were sampled with separate sterile scissors and forceps to prevent cross-contamination of samples. Placental cotyledon, umbilical cord and cord blood were similarly collected aseptically from 1 cow (Herd 1) at parturition. Mismothering was not considered an issue in this study with calving in Herds 1 and 2 occurring over an extended period and calves and their dams being separated from the rest of Herd 3 *post-partum*.

### Packed cell volume, blood and serum preparation

Packed cell volume (PCV) was determined within 4 h of sampling using a haematocrit and is reported as a percentage of the original blood sample. Serum was collected from the clotted blood samples *via* centrifugation for ELISA analysis. EDTA blood samples and sera were frozen at -20 °C until required.

### DNA extraction

DNA was extracted from EDTA blood using a detergent-proteinase K based method (DPK) as previously described [29]. Negative extraction controls were included with each batch of DNA extractions at a minimum of 1 per 19 samples. DNA was purified from tissues using the DNeasy Blood and Tissue Kit (Qiagen, Hilden, Germany) using a final elution volume of 100 μl.

### Quantitative PCR (qPCR)

DNA testing was carried out using a validated multiplex qPCR which includes a universal probe for the detection of all genotypes of *T. orientalis*, along with specific probes for the Ikeda and Chitose genotypes [30]. A qPCR specific for the Buffeli genotype was also carried out for all samples as previously described [31].

### ELISA testing

Serum samples were tested by MPSP ELISA [32] with the following modifications; plates were coated with 1.0 μg antigen and incubated for 1 h on a shaker, wash steps were carried out using TBST (Tris-buffered saline 0.05% Tween 20), the plates were blocked with 1% Bovine Serum Albumin (Roche, Basel, Switzerland) in TBST. Results were expressed as an ELISA ratio (ER: mean OD test serum/mean OD of the negative control serum). Sera with an ER < 2 were considered negative and an ER ≥ 2 as positive for *T. orientalis* MPSP antibodies [32].

### Statistical analysis

Statistical analysis was carried out using Prism 4.0 (GraphPad Software, La Jolla, CA, USA). Spearman’s correlation (two-tailed) was used to determine the relationship between ELISA ratios of cows and calves in Herds 1–3 and at abattoir.

## Results

### qPCR

Of all cows tested, 83.3% (80/96) were qPCR positive with varying parasite loads (Table 1) and *T. orientalis* genotypes (Table 2) observed. Of the 97 newborn (< 48 h old) and foetal calves tested only 2 were qPCR positive (2.1%) (Table 1). Thus, across all samples tested transplacental transmission occurred in 2.5% of cases (2 calves from 80 positive cows).

**Table 1:** Quantitative PCR results for cows and calves

qPCR result	Herd 1		Herd 2		Herd 3	
	TP1		TP1		TP2	
	Cows	Calves	Cows	Calves	Cows	Calves
Negative (*n*)	0	25	0	21	11	30
Low (*n*)	26	0	21	0	10	0
Moderate (*n*)	0	1	0	0	9	0
High (*n*)	0	0	0	0	0	0
Total (*n*)	26	26	21	22	30	30

*Abbreviations*: *TP1* Time point 1 (approximately 5 weeks *pre-partum*), *TP2* Time point 2 (within 48 h *post-partum*)

**Table 2:** Genotypes detected in cows and calves

Genotype detected	Herd 1		Herd 2		Herd 3	
	TP1		TP1		TP2	
	Cows	Calves	Cows	Calves	Cows	Calves
Ikeda (*n*)	0	0	0	0	10	0
Chitose (*n*)	0	0	0	0	1	0
Buffeli (*n*)	0	0	1	0	1	0
Ikeda/Chitose (*n*)	0	0	1	0	2	0
Ikeda/Buffeli (*n*)	0	0	0	0	1	0
Chitose/Buffeli (*n*)	1	0	0	0	1	0
Ikeda/Chitose/Buffeli (*n*)	25	1	19	0	3	0
None (*n*)	0	25	0	22	11	30
Total (*n*)	26	26	21	22	30	30

*Abbreviations*: *TP1* Time point 1 (approximately 5 weeks pre-calving), *TP2* Time point 2 (within 48 h *post-partum*)

### Herd 1

A total of 100 EDTA blood samples were tested from Herd 1. Of these, 52 were bleeds from 26 cow-calf pairs, with the samples from the cows collected on average 5 weeks prior to parturition (TP1). Calf samples were collected at TP2 (within 48 h of parturition). An additional 48 EDTA bloods were from temporal sampling of a subset of 10 calves which were bled on 4–6 occasions *post-partum* between 3 and 72 d of age. All cows in Herd 1 tested positive by qPCR for *T. orientalis* (100% prevalence) at low infection intensity i.e. < 15,000 gene copies/μl of blood (as defined in [30]) at TP1 (Table 1). Of the 26 calves born from the *Theileria*-positive cows, only 1 (< 36 h old) returned a positive qPCR result. The calf had a moderate infection intensity (50,600 gene copies/μl of blood) and both cow and calf tested positive for all three genotypes of *T. orientalis* (Ikeda, Chitose and Buffeli) (Table 2). Ikeda was the dominant genotype in the infected calf while the Chitose genotype was dominant in the cow. While the cow did not display clinical signs of theileriosis at the time of sampling, the calf was noted as lethargic and moderately ataxic. Subsequent temporal sampling of a subset of calves from Herd 1 (*n* = 10), showed first instances of infection at 10–18 d *post-partum*. Parasite load in these calves peaked between Day 38 and Day 55 *post-partum*. At the peak of infection, all calves had high parasite loads, between 1 × 10^6^ and 6 × 10^6^ gene copies/μl blood (Additional file 1: Figure S1).

Placental cotyledon umbilical cord and cord blood were collected from a single cow from Herd 1 which was subclinically infected with *T. orientalis* and had a normal delivery. No gross abnormalities were observed in the placenta. Placental cotyledon from this cow tested positive for *T. orientalis* [Chitose genotype (460 gene copies/mg of tissue)]*.* EDTA blood collected from the same cow was positive for *T. orientalis* (2,970 gene copies/μl of blood). Ikeda, Chitose and Buffeli genotypes were detected in approximately equal quantities in the blood. Samples of umbilical cord and cord blood both tested *T. orientalis* negative.

### Herd 2

A total of 93 blood samples were tested from Herd 2. Of these, 21 samples were from pre-parturient cows (collected at TP1) and 22 were from newborn calves, including one set of twin calves, collected at TP2. All of the cows tested positive for *T. orientalis* (100% prevalence) at low infection intensities. The majority of cows (20/21) harboured the Ikeda genotype (Table 2). No newborn calves tested positive by qPCR at TP2 (Table 1). Temporal sampling of a subset of calves from Herd 2 (*n* = 10) indicated that all 10 calves became qPCR positive between 12-27 d *post-partum*. Parasite load peaked between Day 29 and Day 50 *post-partum* and ranged from 5.9 × 10^5^ to 5.5 × 10^6^ at the peak of infection (Additional File 2: Figure S2).

### Herd 3

A total of 60 EDTA blood samples, collected from 30 calving cows and their calves, were tested from Herd 3 at TP2. Of the cows, 63% (19/30) were positive for *T. orientalis* and had either low or moderate intensity infections of mixed genotype (Tables 1 and 2). No calves from Herd 3 tested qPCR positive.

#### Samples from aborted and stillborn calves

The dead newborn calf sampled from Herd 1 and the stillborn calf from nearby Bungwahl were both qPCR negative for *T. orientalis*. The remaining 3 samples collected from aborted and stillborn calves at the property at Bega were also negative, despite a high number of clinical cases occurring in that herd at the time of sampling. Routine diagnostic (qPCR) testing of cows from the Bega herd revealed 100% prevalence of *T. orientalis* in the herd at that time and all cows had moderate to high parasite loads of mixed genotype (data not shown).

#### *Abattoir samples*

A total of 38 samples, comprising 19 foetal spleen samples and 19 EDTA bloods from the corresponding cows were tested by qPCR. Cows had varying levels of *T. orientalis* infection with 14/19 positive by qPCR. Only one foetal spleen sample tested positive for *T. orientalis* at a low infection intensity (370 gene copies/mg tissue); all other foetal calves tested negative. The positive foetal calf corresponded to a mid-gestation cow with a moderate *T. orientalis* infection (18,400 gene copies/μl). Both cow and foetal calf tested positive for the Ikeda, Chitose and Buffeli genotypes of *T. orientalis* and were linked back to a herd in Wauchope, NSW, within the *Theileria*-endemic area.

### Packed cell volume

The packed cell volumes (PCVs) of cows were within the normal bovine PCV range, with slight variations between each herd. Although still predominantly within the normal range, calf PCV had a larger range both within and between herds (Table 3).

**Table 3:** Range of PCV at time of sampling for each location

PCV	Herd 1		Herd 2		Herd 3	
	TP1		TP1		TP2	
	Cows	Calves	Cows	Calves	Cows	Calves
< 24	0	1	0	2	0	0
≥ 24	26	25	21	20	30	30
Total (*n*)	26	26	21	22	30	30

*Abbreviations*: *TP1* Time point 1 (approximately 5 weeks pre-calving), *TP2* Time point 2 (within 48 h *post-partum*)

#### Herd 1

*Pre-partum* (TP1) cow PCV ranged between 32 and 47% (mean 40%; SEM 0.78). Calf PCV (TP2) ranged from 23 to 46% (mean 37%; SEM 1.14). The qPCR positive calf had a PCV of 45, while the calf with the lowest PCV (23) was qPCR negative. All 10 calves from Herd 1 that were included in the temporal study, experienced a drop in PCV coinciding with the peak in parasite load (Additional file 1: Figure S1), with 3 animals becoming anaemic between Day 38 and Day 50 *post-partum*. Of these 3 calves, 2 subsequently became seropositive (see below).

#### Herd 2

*Pre-partum* cow PCV ranged from 28 to 44% (mean 36.5%; SEM 0.70), whilst calf PCV ranged from 17 to 45% (mean 36.5%; SEM 1.75). A set of twin heifer calves had the lowest PCV at birth; one being in the clinically anaemic range with a PCV of 17 and the other with a PCV of 21. Both of these calves were qPCR negative for *T. orientalis*. All calves included in the temporal study experienced a drop in PCV over the time course coinciding with the peak in parasite load (Additional file 2: Figure S2), with 5 of the 10 becoming anaemic between Day 33 and Day 49 *post-partum*. Of the 5 anaemic calves, 3 subsequently became seropositive (see below).

#### Herd 3

Cows and calves in Herd 3 were both sampled at TP2. Cow PCV ranged between 28 and 44% (mean 37%; SEM 0.72), while calf PCVs were all in the clinically normal range between 24 and 50% (mean 37.5%; SEM 1.18).

#### Abattoir samples

The PCVs of the cows sampled at abattoir were all in the clinically normal range between 30 and 45% (mean 38%; SEM 0.69).

#### *Theileria orientalis* MPSP antibodies

Cows and calves from Herds 1–3 were tested for *T. orientalis* MPSP antibodies by ELISA. Despite 85.7% (66/77) of cows testing qPCR positive at varying levels, only 9.1% (7/77) of cows tested positive for *T. orientalis* MPSP antibodies by ELISA (ER > 2) (Table 4). Twenty out of 78 (25.6%) newborn calves tested positive by ELISA (Table 4); however, only one of these calves was qPCR positive at birth.

**Table 4:** Cow and calf antibody status (ELISA ratio; ER) for each sampling location

Antibody ER	Herd 1		Herd 2		Herd 3	
	TP1		TP1		TP2	
	Cows	Calves	Cows	Calves	Cows	Calves
Positive (ER > 2)	3	10	2	9	2	1
Negative (ER < 2)	23	16	19	13	28	29
Total (*n*)	26	26	21	22	30	30

*Abbreviations*: *TP1* Time point 1 (approximately 5 weeks pre-calving), *TP2* Time point 2 (within 48 h *post-partum*)

#### Herd 1

Despite a high prevalence (100%) of *T. orientalis* in Herd 1 as determined by qPCR, only 3/26 cows sampled prior to calving tested positive for *T. orientalis* MPSP antibodies. In contrast to the cows, 10 calves tested positive for *T. orientalis* MPSP antibodies, including the one qPCR positive calf. The calves corresponding to the 3 ELISA positive *pre-partum* cows tested positive for post-colostral antibodies. The dead newborn calf from Herd 1 tested ELISA negative.

Temporal sampling of 10 calves from Herd 1 demonstrated that post-colostral antibodies were acquired by 5 of the 10 calves within 4 d of birth; however, anti-MPSP antibodies were no longer detectable in any of these calves by Day 31. Two of these 5 calves originally testing ELISA positive returned to seropositivity between Days 50 and 67 (e.g. Calf 3, Additional file 1: Figure S1), following the peak in parasite load.

#### Herd 2

Of the 21 cows tested, only 2 were ELISA positive, with both of these cows having low infection intensities. In contrast, 9 calves tested ELISA positive. The two positive cows (ER 2.13 and 2.27) had (respectively) an ELISA positive calf (ER 3.52) and an ELISA negative (ER 0.93) calf. Three of the 10 calves included in the temporal study tested positive for post-colostral antibodies within 4 d of birth; however, all 3 calves became seronegative by Day 28 *post-partum*. Five of the 10 calves (only one of which showed prior evidence of post-colostral antibodies) became seropositive for *T. orientalis* MPSP antigen between Day 36 and Day 50 *post-partum*, following the peak in parasite load (Additional file 2: Figure S2).

#### Herd 3

Of the 30 cows tested, 2 were ELISA positive at parturition and both animals had moderate infection intensities. Only one calf tested positive for MPSP antibodies and this calf corresponded to one of the antibody-positive cows.

No significant correlation (*P* > 0.05) was observed between the ERs of cows and calves in Herds 1 or 2; however a significant positive correlation was observed between the ER of cows and calves from Herd 3 (Table 5).

**Table 5:** Correlations between cow and calf ELISA ratios (two-tailed Spearman’s correlation)

Herd	Sampling time point	Correlation between	Spearman’s rho	*P*-value
1	TP1	Pre-parturient cow/calf at birth	0.26	> 0.05
2	TP1	Pre-parturient cow/calf at birth	0.06	> 0.05
3	TP2	Cow/calf at birth	0.50	0.01

*Abbreviations*: *TP1* Time point 1 (approximately 5 weeks pre-calving), *TP2* Time point 2 (within 48 h *post-partum*)

## Discussion

Both paired cow-calf samples and cow-foetal samples from different herds within the south-eastern Australian *T. orientalis* endemic zone were examined in this study. The detection of *T. orientalis* in newborn or foetal calves albeit at a low rate (2 from 80 confirmed qPCR positive cows; 2.5%), is suggestive of transplacental transmission of the parasite from cow to calf. While the presence of *T. orientalis* has been demonstrated at low levels in the colostrum of infected cows [21], this was not considered a likely route of transmission in this study due to the very young age of the calves (< 48 h old). While the majority of the calves examined in this study had access to colostrum from infected mothers at the time of sampling, the detection of *T. orientalis* in a mid-term foetal calf sampled at abattoir precludes the possibility of colostral transfer in that instance. Furthermore, the low levels of *T. orientalis* reported in the colostrum of cows relative to whole blood [21] are unlikely to be sufficient to result in the moderate infection intensity observed in the 36 h old calf from Herd 1. The infections observed in both the calf and the pre-term foetus observed in this study are therefore attributed to transplacental transmission of the parasite. Indeed, transplacental transmission has been detected for a number of other *Theileria* spp. including *T. equi* [22] *T. lestoquardi* [24] and *T. annulata* [25] using molecular methods.

This study is the first report of transplacental transmission of *T. orientalis* under field conditions using molecular techniques, although prior microscopic observations suggested transplacental transmission may occur in Australian cattle [10]. In that study, a calf sampled at 4 days of age returned a *Theileria*-positive blood smear. The rate of transplacental transmission determined in the current study is comparable to a Japanese study in which approximately 5% of calves sampled at 1–2 days of age were deemed positive for *T. orientalis* upon blood film examination. The parasitaemia reported in those calves was very low (0.01–0.06%) [27]. Similarly, the qPCR positive foetus detected in this study also had very low levels of *T. orientalis* in the spleen (370 gene copies/mg of tissue). Interestingly, a moderate intensity infection was detected in the qPCR positive calf from Herd 1 and this calf also displayed clinical signs of theileriosis *post-partum* (lethargy and moderate ataxia). Clinical signs associated with transplacental transmission have also been described previously for *T. annulata* [25, 33]. In both cases of transplacental transmission detected in this study, the calves were found to be infected with the Ikeda, Chitose and Buffeli genotypes of the parasite, suggesting that all three genotypes can traverse the placental barrier. The presence of the pathogenic Ikeda genotype in these calves suggests that transplacental transmission has the potential to contribute to the persistence of clinical theileriosis within herds given that this genotype has specifically been associated with major outbreaks of disease in Asia and Australasia [1–4, 6].

The relatively low rate of transplacental transmission observed in this study, under field conditions, is in contrast to a previous study in which 6 cows experimentally-infected with *T. orientalis* produced 100% incidence of infection in calves, including two aborted animals [26]. In that study, the cows were naïve to *T. orientalis* and transmission was achieved with a large burden of ticks (200/cow) which had been artificially fed on a highly parasitaemic animal. In contrast, the majority of cow-calf pairs in this study were from *T. orientalis*-endemic areas, where a high prevalence of infection had already been established. It has been noted previously that naïve cattle are more susceptible to clinical theileriosis [2, 7] and that cattle with clinical theileriosis have a higher parasite burden than subclinically-infected animals [30]. If transfer of individual *T. orientalis* parasites across the placenta is a relatively rare event, then the likelihood of this mode of transmission would be expected to increase when the cows have high infection intensities, as has been described for transplacental transmission of *Plasmodium falciparum* in humans [34]. It was noteworthy therefore, that the opportunistic samples collected from aborted and stillborn calves on a property near Bega, NSW which was experiencing a clinical theileriosis outbreak, and in which the cows had moderate-high parasite loads, tested negative for *T. orientalis*. This suggests that infection of the calf *in utero* may not be the major cause of theileriosis-related abortions. Infection of pregnant cows with the apicomplexan parasite *Neospora caninum* also frequently results in calf abortion. While *N. caninum* is readily transmitted transplacentally causing foetal infection [35], other proposed mechanisms of abortion are the proliferation of the parasite in the placenta causing placental lesions and subsequent calf hypoxia or restriction of nutrition to the foetus. The one placenta examined in this study appeared grossly normal, but was derived from a subclinically infected animal with a low parasite load. Furthermore, the placental cotyledons tested qPCR positive, but both cord blood and calf tested negative suggesting that the placental barrier was not compromised in this instance [36]. Other potential mechanisms of abortion in *N. caninum* include immunological imbalances in the placenta resulting in a harmful inflammatory cytokine response against the foetus, or placental inflammation and prostaglandin release inducing abortion or foetal damage [37]. Similar mechanisms may be responsible for abortion due to *T. orientalis* when the infections are severe. Maternal anaemia may also be a contributing factor to foetal damage through calf hypoxia in these instances.

High tick burdens, as described in the Baek et al. experimental infection study [26], may explain the high rate (100%) of transplacental transmission detected in that study. Ticks are known to produce an immunosuppressive effect in the host which may be expected to lead to increased parasite burden [38]. Cows examined in this study were not noted to be infested with large numbers of ticks. Furthermore, it is unclear which life-cycle stage(s) of *T. orientalis* are able to cross the bovine placenta; however both the sporozoite and schizont phases are transient in the cow, precede the formation of intraerythrocytic piroplasms, and are dependent upon transfer *via* the tick vector. Therefore transplacental transfer of the sporozoite or schizont life-cycle stages would require active infection of pregnant cows *via* ticks during gestation. Conversely, transplacental transfer of the piroplasm stage of the parasite would contribute to the persistence of *T. orientalis* in cattle herds during seasons where ticks are inactive or sparse.

The mechanism of transplacental transmission of *T. orientalis* was not established in this study, but is worthy of further investigation. Cattle, like other ruminants, have an epitheliochorial placenta and this “non-invasive” form of placentation is believed to have evolved under selective pressure from parasites to form a tight barrier against foetal infection [39]. Furthermore, studies of *T. equi* infection in horses, which also display epitheliochorial placentation, have suggested that transplacental transmission occurs relatively early in gestation, before the placenta is fully formed [40]. It was noteworthy therefore, that in this study *T. orientalis* infection was detectable in a mid-term foetal calf, suggesting that transplacental transmission occurs in the earlier stages of pregnancy.

Epitheliochorial placentation also precludes prenatal transfer of maternal antibodies from cow to calf, which are instead acquired from the colostrum. The qPCR positive calf from Herd 1 had a moderate infection intensity 36 h post-birth, and was also seropositive; however it is unclear whether these antibodies resulted from a foetal immune response to infection *in utero* or were maternally derived *post-partum*. Interestingly, very few cows from Herds 1 and 2, in which *T. orientalis* was endemic and infections were subclinical, were MPSP seropositive. These findings are consistent with a prior study demonstrating much higher seroconversion rates in clinically affected relative to subclinically infected animals [32]. The ERs of cows and calves in Herd 3, but not Herds 1 and 2 were significantly correlated, which likely reflects the sampling time point used for cows in Herd 3 (within 48 h of birth) compared to Herds 1 and 2 (5 weeks *pre-partum* on average). In Herds 1 and 2, more seropositive calves than seropositive cows were detected and this may be explained by the increased concentration of antibodies in colostrum delivered to the calves [41]. The seropositive status of calves was nonetheless short-lived, with calves in the temporal study testing seronegative before 4–4.5 weeks *post-partum*. Whether antibodies are protective against *T. orientalis* is currently unclear; however, the peak in parasite load in all calves tested occurred after the decline in antibody levels, at 5–8 weeks *post-partum*. Notably, very high parasite loads were observed in calves at this age, with some calves producing an adaptive immune response within 1–2 weeks of the peak infection intensity and in some cases coinciding with the onset of anaemia. The time frame within which *T. orientalis* was first detected in the calves by qPCR (10–27 days) is comparable with prior studies on naïve adult cattle introduced to a *T. orientalis*-affected herd [31] and is consistent with vector transmission [26, 42].

## Conclusions

In conclusion, we demonstrate that transplacental transmission can occur in cattle herds and contribute to persistence of *T. orientalis*, but occurs at a relatively low rate. Infection *in utero* does not appear to be a direct cause of abortion, but may instead be dependent on the maternal clinical state. Despite the relatively low rate of transplacental transmission of *T. orientalis*, calves experience a high rate of infection in the first weeks of life with time to patency consistent with vector transmission. Acquisition of maternal antibodies can occur but is short-lived with some calves producing an adaptive immune antibody to *T. orientalis* post-infection.

## Additional files

Additional file 1: Figure S1. Corresponding parasite load (qPCR), ER (MPSP ELISA) and PCV data derived from 4 representative calves from the Herd 1 temporal study. A marked increase in parasite load co-incides with a decrease in PCV in all calves with calf 3 becoming anaemic on Day 50 *post-partum*. Two of the four calves tested positive for maternal antibodies *post-partum* but subsequently tested negative. Calf 3 appeared to mount an adaptive serological response to *T. orientalis* following the peak in parasite load. (DOCX 127 kb)

Additional file 2: Figure S2. Parasite load (qPCR), ER (MPSP ELISA) and PCV data derived from 4 representative calves from the Herd 2 temporal study. A marked increase in parasite load coincided with a decline in PCV, with calves 2–4 becoming anaemic between Day 40–50. Two of the four calves shown tested positive for maternal antibodies *post-partum*. Calves 2–4 appeared to mount an adaptive serological response following the peak in infection intensity. (DOCX 141 kb)

## Abbreviations

ELISA: enzyme-linked immunosorbent assay
ER: ELISA ratio
PCV: packed cell volume
qPCR: quantitative polymerase chain reaction
TP1: time point 1
TP2: time point 2
