# Supplementary material for: Transplacental transmission of Theileria orientalis occurs at a low rate in field-affected cattle: infection in utero does not appear to be a major cause of abortion
Source: Parasit Vectors. 2017 May 8;10:227. doi: 10.1186/s13071-017-2166-9 (PMC5423014; doi:10.1186/s13071-017-2166-9)
Supplement: Supplementary file 1 — Corresponding parasite load (qPCR), ER (MPSP ELISA) and PCV data derived from 4 representative calves from the Herd 1 temporal study. A marked increase in parasite load co-incides with a decrease in PCV in all calves with calf 3 becoming anaemic on Day 50 post-partum. Two of the four calves tested positive for maternal antibodies post-partum but subsequently tested negative. Calf 3 appeared to mount an adaptive serological response to T. orientalis following the peak in parasite load. (DOCX 127 kb) [file 13071_2017_2166_MOESM1_ESM.docx]

Calf 1

Calf 2

Calf 3

Calf 4

Additional File 1. Corresponding parasite load (qPCR), ER (MPSP ELISA) and PCV data derived from 4 representative calves from the Herd 1 temporal study. A marked increase in parasite load co-incides with a decrease in PCV in all calves with calf 3 becoming anaemic on Day 50 post-partum. Two of the four calves tested positive for maternal antibodies post-partum but subsequently tested negative. Calf 3 appeared to mount an adaptive serological response to *T. orientalis* following the peak in parasite load.
