# Supplementary material for: Transplacental transmission of Theileria orientalis occurs at a low rate in field-affected cattle: infection in utero does not appear to be a major cause of abortion
Source: Parasit Vectors. 2017 May 8;10:227. doi: 10.1186/s13071-017-2166-9 (PMC5423014; doi:10.1186/s13071-017-2166-9)
Supplement: Supplementary file 2 — Parasite load (qPCR), ER (MPSP ELISA) and PCV data derived from 4 representative calves from the Herd 2 temporal study. A marked increase in parasite load coincided with a decline in PCV, with calves 2–4 becoming anaemic between Day 40–50. Two of the four calves shown tested positive for maternal antibodies post-partum. Calves 2–4 appeared to mount an adaptive serological response following the peak in infection intensity. (DOCX 141 kb) [file 13071_2017_2166_MOESM2_ESM.docx]

Calf 3

Calf 1

Calf 4

Calf 2

Additional File 2. Parasite load (qPCR), ER (MPSP ELISA) and PCV data derived from 4 representative calves from the Herd 2 temporal study. A marked increase in parasite load coincided with a decline in PCV, with calves 2-4 becoming anaemic between Day 40-50. Two of the four calves shown tested positive for maternal antibodies post-partum. Calves 2-4 appeared to mount an adaptive serological response following the peak in infection intensity.
